# Supplementary material for: Polyvinylidene Fluoride-Graphene Oxide Membranes for Dye Removal under Visible Light Irradiation
Source: Polymers (Basel). 2020 Jul 7;12(7):1509. doi: 10.3390/polym12071509 (PMC7407290; doi:10.3390/polym12071509)
Supplement: Supplementary file 1 [file polymers-12-01509-s001.pdf]

Supporting Information

Polyvinylidene fluoride-graphene oxide membranes for dye removal under visible light irradiation

Sabri Alyarnezhad[1], Tiziana Marino[2,*], Jalal Basiri Parsa[1], Francesco Galiano[2], Claudia Ursino[2], Hermenegildo Garcìa[3], Marta Puche[3], Alberto Figoli[2,*]

[1] Department of Applied Chemistry, Faculty of Chemistry, Bu-Ali Sina University, Hamedan 65174, Iran
[2] Institute on Membrane Technology, ITM-CNR, Via P. Bucci 17c, 87030 Rende (CS), Italy
[3] Instituto Universitario de Tecnología Química (CSIC-UPV), Universidad Politecnica de Valencia, AV. de los Naranjos s/n, 46022, Valencia, Spain

* Correspondence: a.figoli@itm.cnr.it, tel. 0039 0984 492027; t.marino@itm.cnr.it

Total number of pages (including cover page): 6

Total number of Equations: 4

Total number of Figures: 3

Characterization of GO. Combustion chemical analysis indicates that the carbon and sulphur content of the material was 54 and 4.2 %, respectively. Manganese and iron content determined by ICP-optical emission spectroscopy (ICP-OES) was 160 and 10 ppm, respectively. TEM images of GO were taken using a Philips CM300 FEG microscope operating at 100 kV. The Raman measurements were carried out in a Renishaw Raman spectrophotometer coupled with a Leica optical microscope. Raman spectra were recorded at room temperature using an Ar ion laser (514.5 nm) as an excitation source. XRD patterns were measured with a Philips X′Pert diffractometer using CuKα radiation (1.54118 Å). XPS analyses were performed using a VG-Escalab 210 photoelectron spectrometer with a monochromatic MgKα X-ray source. AFM images were recorded by using a Multimode Nanoscope 3A instrument operating in tapping mode with a Si wafer as substrate. FTIR spectroscopy was conducted by using a Nicolet 8700 Thermo spectrometer.

Characterization of the membranes

Scanning Electron Microscopy. The morphology of cross section, top and bottom-side of the prepared membranes was analyzed by SEM (Zeiss EVO, MA100, Assing, Italy). Cross-sections were prepared by freeze fracturing the samples in liquid nitrogen. Prior the analyses, all the samples were sputter coated with a thin layer of gold in order to make them conductive.

Atomic Force Microscopy. AFM was used in order to study the effects of additives and GO loading on membrane surface morphology and roughness. The AFM device was a Bruker Multimode 8 with Nanoscope V controller. Data were acquired in tapping mode, using silicon cantilevers (model TAP150, Bruker, Durham, UK). GO membranes surfaces were mounted on a holder stub using double-sided scotch tape and they were imaged in a scan size of 2 µm x 2 µm.

Contact angle measurement. Water contact angle (CA), which indicates the wettability of a membrane, was measured by means of a CAM100 instrument (Nordtest srl, GI, Serravalle Scrivia (AL) Italy). For this purpose, a water droplet was dropped on the surface of a membrane sample, located on a suitable support, by using a micro-syringe and the contact angle was then measured. The procedure was repeated four times for each membrane and the average value was considered.

Porosity. Membrane porosity is defined as the void fraction inside the membrane relative to the total volume of the membrane. For measuring overall porosity (ε) of the membranes, membrane samples were measured dried and after soaking them in kerosene for 24 hours. The dry and wet weights of the samples were recorded. The porosity of the membranes was calculated according to the following equation[1]:

$$\varepsilon = \frac{(\frac{W_w - W_d}{\rho_k})}{(\frac{W_w - W_d}{\rho_k}) + (\frac{W_d}{\rho_p})} \times 100 \qquad (1)$$

Where W_w (g) is the weight of the wet membrane, W_d (g) is the weight of dry membrane, ρ_k is the density of kerosene (0.82 g /cm^3), and ρ_p is the density of the polymer (1.75 g /cm^3).

Pore size. Mean pore size, largest pore size and bubble point pressure for the membranes were determined by using PMI Capillary Flow Porometer (CFP 1500, AXEL USA, Porous Materials Inc.). The operating mode used for the calculation of parameters was wet-up/dry-up. Flourinert^{TM} (FC-40) (from Sigma-Aldrich, Bollate, Italy) with surface tension 16×10^{-3} N m^{-1} was used as a wetting liquid. Next, the sample was clamped on a holder and nitrogen gas was applied to one side of the membrane with a certain increasing pressure gradient. Nitrogen was forced to pass through the membrane pores by replacing Flourinert^{TM} and the membrane pore radius was calculed by Laplace equation[2]:

$$r_p = \frac{2\sigma}{\Delta P}\cos\theta \qquad (2)$$

Where r_p is the radius of the pore (m), σ is surface tension at the interface of liquid and air, ΔP is membrane pressure difference (Pa), and θ is contact angle (°). For analyzing the data, Capwin software was applied. For each membrane two measurements were performed and the results of each test were imported as an excel file and average value with related standard deviations were reported.

Mechanical properties. The mechanical properties of the membrane including tensile stress, Young's modulus and elongation at break were measured by tensile testing machine, Zwick/Roell Z2.5 (Genova, Italy). At least five samples (10 x 50 mm) for each membrane were analyzed.

Pure Water Permeability. Permeability tests of the membranes were performed using a cross flow filtration apparatus with constant stirring and 1.54 cm^2 effective area. Permeate flux, J_{w1} $\left(\frac{kg}{m^2 h\ bar}\right)$, and removal efficiency R (%), were calculated according to equations (3) and (4), respectively:

$$J_{w1} = \frac{M}{A\Delta tP} \qquad (3)$$

$$R\ (\%) = \left(1 - \frac{C_p}{C_f}\right) \times 100 \qquad (4)$$

Where M is the weight of collecting permeates (kg), A is membrane operative area (m^2), Δt is permeation time (h) and P (bar) is applied pressure. The performance of the membranes was evaluated

by degradation of MB^+ dye. Removal efficiency R (%) was calculated using equation (4), where C_p and C_f were the concentrations of the dye (mg/L) in permeate and feed, respectively.

Figures:

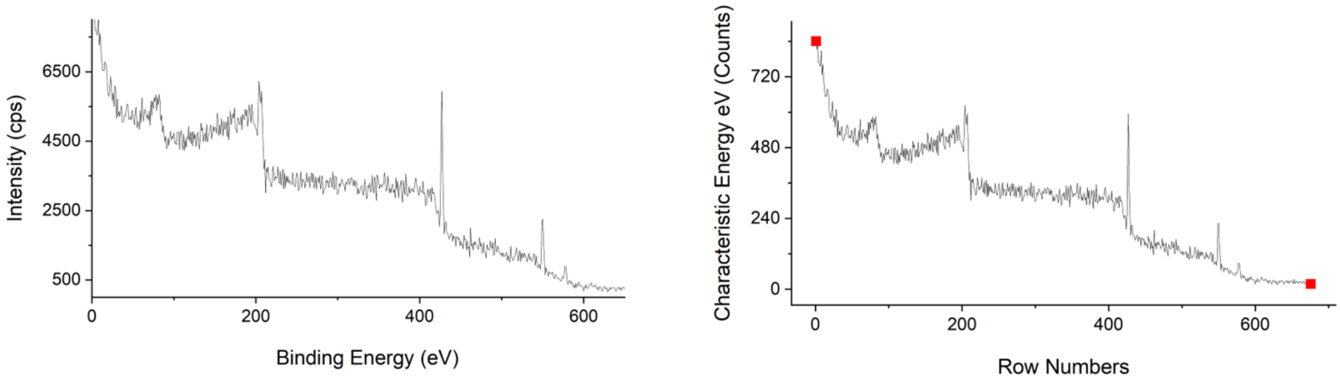

Figure S1. XPS survey of the two GO samples. The peaks corresponding to C1s and O1s appear in the high resolution XPS between 283 and 290 and 536 and 539eV, respectively. For the high resolution C1s peak see Figure 2d.

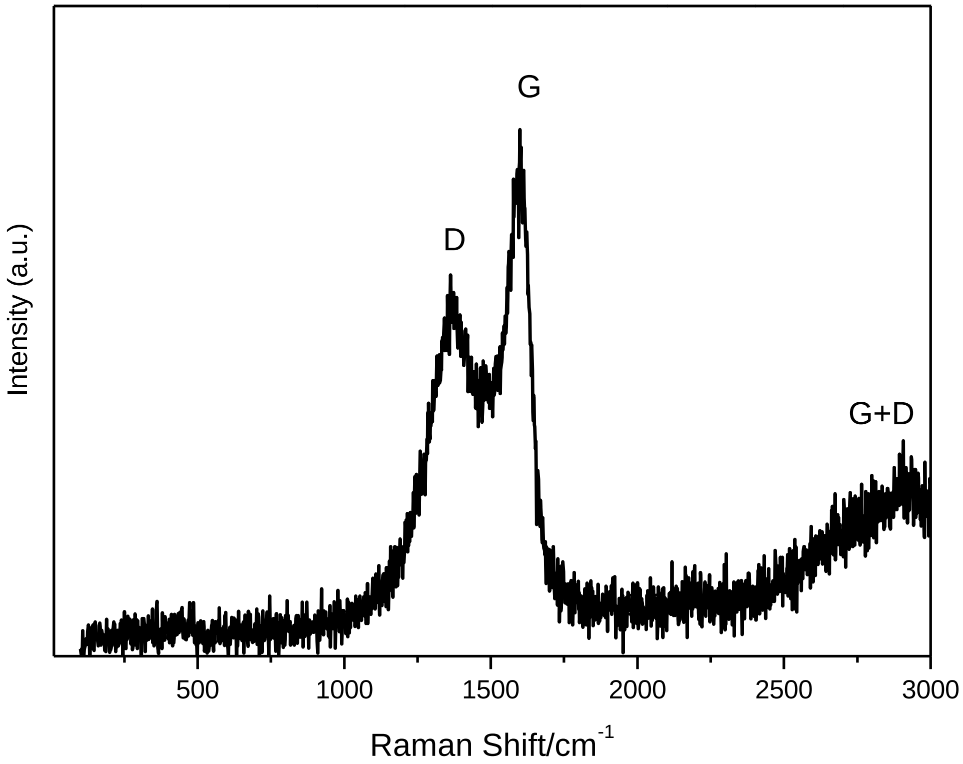

Figure S2. Raman spectrum of GO

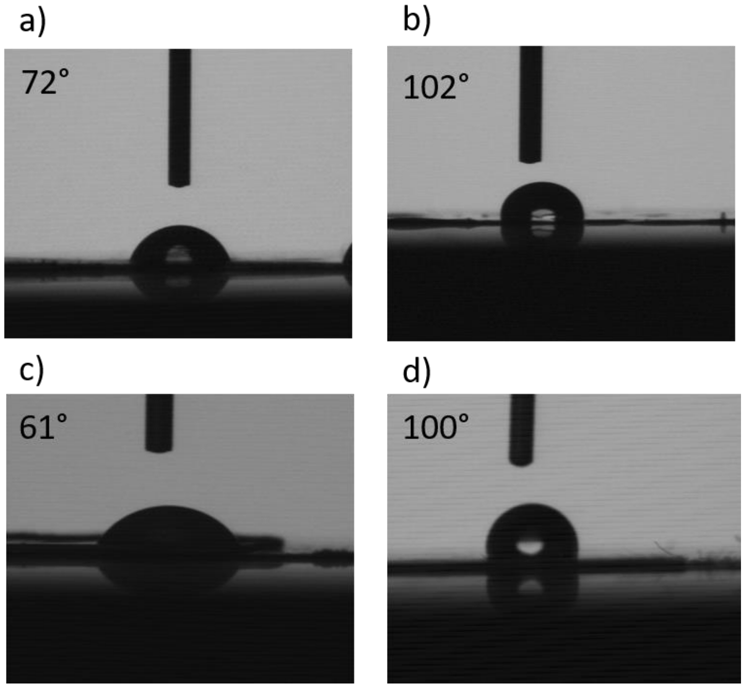

Figure S3. CA pictures of M1 (a, b) and M7 (c, d) membranes.

Top surface (a, c), bottom surface (b, d)

References

(1) Sukitpaneenit, P.; Chung, T. S. Molecular Elucidation of Morphology and Mechanical Properties of PVDF Hollow Fiber Membranes from Aspects of Phase Inversion, Crystallization and Rheology. *J. Memb. Sci.* **2009**. https://doi.org/10.1016/j.memsci.2009.05.029.

(2) Jena, A.; Gupta, K. Advances in Pore Structure Evaluation by Porometry. *Chemical Engineering and Technology*. **2010**. https://doi.org/10.1002/ceat.201000119.
